# Breast Implant Illness (BII) As a Clinical Entity: A Systematic Review of the Literature

**DOI:** 10.1093/asjof/ojae007.073

**Published:** 2024-04-12

**Authors:** Kshipra Hemal, Raeesa Kabir, Eloise Stanton, Thomas Sorenson, Carter Boyd, Nolan Karp, Mihye Choi

**Affiliations:** NYU Langone Health, New York, NY; University of Minnesota, Minneapolis, MN; University of Southern California, Los Angeles, CA; NYU Langone Health, New York, NY; NYU Langone Health, New York, NY; NYU Langone Health, New York, NY; NYU Langone Health, New York, NY

## Abstract

**Goals/Purpose:**

Breast implant illness (BII) has become a contentious subject in recent years, raising concerns among both patients and healthcare professionals. While some studies have reported associations between breast implants and autoimmune diseases, others have failed to establish a definitive link. Therefore, the objective of this systematic review is to provide a comprehensive evaluation of the existing literature with a specific emphasis on identifying any symptom or patient patterns to critically evaluate the existence of BII as a distinct entity.

**Methods/Technique:**

A comprehensive search of relevant published studies up until 2023 was conducted across multiple databases, including PubMed and MEDLINE. A total of 31 studies met inclusion criteria in our analysis. To qualify for inclusion, studies had to focus on breast implant illness and associated systemic symptoms. Two reviewers independently assessed the abstracts, manuscripts, and extracted data from the selected papers. From the included studies, cohort size, reason for implantation, mean age, implant type, implant texture, plane of implant placement, mean follow-up time, implant explantation status, time to implant explantation, symptom resolution after explantation, infections, and complications were extracted. Descriptive statistics was used where appropriate.

**Results/Complications:**

The mean age of patients was 44.2 ± 9.30 years for all studies included. For studies that included length of time between implant exposure and onset of clinical symptoms (9/31; 29.0%), the mean time from implant or biomaterial exposure to onset of clinical symptoms of BII was 13.4 ± 2.92 years. Fourteen (14/31; 45.2%) studies reported implant explantation status with 60% of the total patient population choosing to remove their implants. Among these, 9 studies reported symptom improvement in 657 patients (83.5%) from a total of 788 patients undergoing implant explantation. Eight studies (8/31; 25.8%) reported whether patients experiencing BII related symptoms were in the cosmetic or reconstructive group. Patients in the cosmetic cohort (899/1005; 89.5%) experienced significantly more BII-related symptoms compared to patients in the reconstructive cohort (213/352; 60.5%) (p < 0.001).

**Conclusion:**

This systematic review provides a comprehensive overview of the current state of knowledge regarding BII. While the literature offers valuable insights into the potential associations and outcomes related to BII, there are several limitations stemming from heterogeneity in study designs, patient populations, and reporting practices. Our study highlights a relationships between BII and indication for implants (cosmetic vs. reconstructive), infection, and explantation, among other variables, offering valuable directions for future research.

